# Report of Committee on Nomenclature and Scientific Investigation in Periodontology

**Published:** 1917-11

**Authors:** 


### REPORT OF COMMITTEE ON NOMENCLATURE AND SCIENTIFIC INVESTIGATION IN PERIODONTOLOGY

### NOMENCLATURE

*Oral Hygiene*—That branch of hygiene dealing with the mouth.

*Oral Prophylaxis*—A term applied to any procedure designed to prevent diseased conditions of the teeth and periodontal tissues and to protect and preserve the general health. Such procedure may be instructional, orthodontic, surgical, mechanical or medicinal.

*Oral Prophylactic Treatment*—A systematized periodic treatment designed to maintain the health of the teeth and periodontal tissues.

*Periodontoclasia*—The process of destruction of the tissues immediately surrounding the teeth.

*Periodontology*—The study of periodontoclasia.

*Periodontal or Periodontic*—Of or pertaining to the tissues immediately surrounding the teeth.

*Periodontia*—That branch of dentistry devoted to treating abnormal conditions of periodontal tissues.

*Periodontist*—A dentist who practices periodontia as a specialty.

### CLASSIFICATION OF PERIODONTAL DISEASES

*Recession*—Atrophy of the gum margin and underlying tissues, resulting in exposure of the cementum.

*Gingivitis*—Inflammation of the gum margin.

*Acute Ulcerative Gingivitis*—Acute gingivitis, producing rapid superficial ulceration of the gum margin.

*Suppurative Periodontoclasia*—Separation of the principal tissues from the cementum with macroscopic formation of pus.

*Nonsuppurative Periodontoclasia*—Separation of the periodontal tissues from the cementum without the macroscopic formation of pus.

*Alveolar Periodontoclasia*—Resorption of the alveolar process without separation from the cementum and without macroscopic pus formation.

*Periodontal Abscess*—A periodontal abscess not primarily involving the periapical tissues.

### PERIODONTOCLASIA

### ETIOLOGICAL FACTORS

*Favoring Disease:*

1. Abnormal tooth surface.
    - Soft deposits.
    - Hard deposits.

Denuded cementum.
Abnormal tooth form.

2. Malocclusion.
   Defective contacts.
   Traumatic occlusion.
3. Defective operative and prosthetic procedures.
   Defective contacts.
   Defective occlusal restorations.
   Defective margins.
4. Abnormal systemic conditions.
   Auto intoxication.
   Syphilis, Diabetes, etc.
   Nutritional disturbances.
5. Parasitic invasions.
   Bacteria.
   Protozoa.
6. Trauma.
   Improper brushing.
   Clamps, ligatures, etc.
   Bloes, etc.

ETIOLOGICAL FACTORS

may be either

| PRIMARY | SECONDARY |
| --- | --- |
| Abnormal tooth surface. | Abnormal tooth surface. |
| Malocclusion. | Malocclusion. |
| Defective operative and prosthetic procedures. | Abnormal systemic conditions. |
| Trauma. | Parasitic invasion. |

